# Work Stress in NHS Employees: A Mixed-Methods Study

**DOI:** 10.3390/ijerph17186464

**Published:** 2020-09-04

**Authors:** Jermaine M. Ravalier, Andrew McVicar, Charlotte Boichat

**Affiliations:** 1 Psychology Centre for Health and Cognition, Bath Spa University, Newton Park Campus, Newton St Loe, Bath BA2 9BN, UK; 2 Faculty of Health, Education, Medicine and Social Care, Anglia Ruskin University, Bishop Hall Lane, Chelmsford CM1 1SQ, UK; Andy.Mcvicar@anglia.ac.uk; 3 University of the West of England, Frenchay Campus, Coldharbour Lane Bristol, Bristol BS16 1QY, UK; Charlotte.Boichat@uwe.ac.uk

**Keywords:** work stress, working condition, communication, peer support, wellbeing, mixed methods

## Abstract

The United Kingdom’s National Health Service (NHS) has a higher-than-average level of stress-related sickness absence of all job sectors in the country. It is important that this is addressed as work stress is damaging to employees and the organisation, and subsequently impacts patient care. The aim of this study was to gain an in-depth understanding of working conditions and wellbeing in NHS employees from three employing NHS Trusts through a mixed-methods investigation. First, a cross-sectional organisational survey was completed by 1644 respondents. Questions examined working conditions, stress, psychological wellbeing, job satisfaction, and presenteeism. This was followed by 33 individual semistructured interviews with NHS staff from a variety of clinical and nonclinical roles. Quantitative findings revealed that working conditions were generally positive, although most staff groups had high levels of workload. Regression outcomes demonstrated that a number of working conditions influenced mental wellbeing and stress. Three themes were generated from thematic analysis of the interview data: wellbeing at work, relationships, and communication. These highlight areas which may be contributing to workplace stress. Suggestions are made for practical changes which could improve areas of difficulty. Such changes could improve staff wellbeing and job satisfaction and reduce sickness absence.

## 1. Introduction

The United Kingdom’s (UK) National Health Service (NHS) is the single biggest employer of over 1.5 million individuals in the country and is funded primarily by taxes. The NHS provides healthcare to UK citizens which is free at the point of delivery. However, employing NHS Trusts are largely running a financial deficit due to the UK government’s austerity agenda of recent years, which has meant lower levels of NHS funding [1], with these financial deficits subsequently affecting staff and being a distinct barrier toward implementing wellbeing initiatives for NHS staff [2], but also care provided to patients [3]. Indeed, the health and social care occupational sectors now have the highest levels of stress-related sickness absence in the country, estimated to be 46% higher than the UK average [4]. Approximately 40% of all staff sickness absence in the NHS is due to work stress [5] and costs the NHS up to £400 million per year [6]. This project presents the outcomes of a mixed-methods study looking at stress in the NHS and strategies which can be implemented in order to improve staff experience.

### 1.1. Stress in the Workplace

Stress in the workplace is defined by the United Kingdom Health and Safety Executive (HSE) as “the adverse reaction people have to excessive pressures or other demands placed on them” [7]. The job demands–resources (JDR; [8]) model is one of the most widely applied theories of work stress. It proposes that employees encounter job demands, which require physical or psychological effort such as high work pressure or problems related to reorganisation. Conversely, job resources (such as support from peers, feedback, or career opportunities) are aspects which are positive in terms of reducing demands, helping to achieve goals, or may be important for personal development. The model suggests that resources can act as a buffer against job demands, but if job demands outweigh the resources available, this can lead to stress and eventually burnout and absenteeism [9,10].

The HSE reports that over 15.4 million working days were lost due to stress, depression, and anxiety in 2017–2018, accounting for 57% of all days lost at work due to sickness absence [11]. Indeed, stress and mental ill health combined are the biggest cause of long-term sickness absence (i.e., that which lasts 4 weeks or more) in the UK, and is second only to colds and flu in terms of short-term absences [12]. The Health and Social Care sector has the highest level of sickness absence of all employment sectors in the UK [11]. Within the NHS, the five-year forward view argues strongly that mental health and wellbeing should be given equal priority statement as physical health in terms of funding and staffing. Indeed, staff health and wellbeing is such an important topic in the NHS that it is linked to the levels of funding awarded to Trusts through the Health and Wellbeing Commissioning for Quality and Innovation (CQUIN).

Consequently, in a bid to support organisations in dealing with stress in the workplace, the HSE released a set of management standards in 2004 [13]. The HSE management standards approach argues that should employees work under poor working conditions (or psychosocial hazards) for an extended period of time, this can lead to sickness absence [13]. In particular, seven hazards (demands, control, managerial support, peer support, relationships, role, and change) are discussed, with organisational evaluation of these hazards possible by distributing the associated Management Standards Indicator Tool (MSIT) [14]. Poor levels of these hazards in the public sector have been shown to be related to organisational outcomes such as sickness presenteeism [15], intentions to leave the profession, and job dissatisfaction [16].

### 1.2. Impact of Stress on Health and Patient Care

Both persistent (chronic) and acute stress have been demonstrated to impact employee psychological and physiological health, although various researchers have suggested that it is generally chronic stress which may lead to stress, anxiety, and depression sickness absence, as well as physiological illness [13]. For example, the INTERHEART studies [17] demonstrated that chronic work stress is as much of a risk factor for the development of cardiovascular disease as well-known problems such as high blood pressure and smoking. Similarly, chronic stress is related to the development of metabolic syndrome, which is a risk factor for the development of Type 2 Diabetes [18].

Furthermore, research is continually demonstrating that for those working in patient-facing roles, positive levels of mental health is generally associated with providing better quality care to their patients [19]. In social work, for example, high levels of turnover and sickness absence have been linked to poor working conditions, subsequently meaning more inefficient care and higher costs in terms of replacement staff [20]. Similarly, West and Dawson [21] demonstrated that positive levels of employee engagement in the NHS was linked to better levels of organisational outcomes such as absenteeism and turnover and patient-related outcomes such as satisfaction, mortality, and infection rates.

### 1.3. The Present Study

Understanding the key stressors, and approaches which can be undertaken in order to reduce stress and thus improve engagement, in NHS employees are key considerations for individual employees, employing Trusts, and the patients they work with. The aim of this research, therefore, is to undertake a mixed-methods study into the working conditions faced by NHS staff in three large employing Trusts, the impact that these hazards have on employee wellbeing, and strategies which can be implemented by the employing healthcare organisation to maintain positive and improve wellbeing for healthcare staff.

## 2. Materials and Methods

### 2.1. Methods and Participants

A mixed-methods study, consisting of an employee survey and individual semistructured interviews, was conducted to investigate the influence of working conditions on stress and wellbeing in NHS staff employed in one of three NHS Trusts in the southwest of the UK. A mixed methods approach involving quantitative surveys and qualitative semistructured interviews was undertaken because it can allow in-depth exploratory and confirmatory research in health and social care settings [22]. Mixed-methods approaches can also be used with health and social care research in order to develop and understand improvement processes [23]. This mixed-methods approach therefore allowed the exploration of working conditions and health and wellbeing through a quantitative survey, and following qualitative interviews allowed an in-depth exploration of these findings in addition to the development of improvement mechanisms with health and social care organisations.

Quantitative survey data was collected using the web-based data collection software Qualtrics (www.qualtrics.com), with the research team putting together an email containing the survey link which was sent to all staff (both clinical and nonclinical) in the three Trusts by the wellbeing lead in each Trust, on behalf of the research team. Links were sent to staff in February 2018, a reminder sent two weeks later, and the data collector closing one week following. The first page of the online survey was an informed consent form, and the survey completed with a full debrief form.

Individuals were invited to qualitative semistructured interviews via another all-staff email designed by the research team and sent by senior management in each Trust. Interviews were conducted on a first-come first-served basis, with iterative rounds of data collection and analysis continuing through to saturation point in which no new themes emerged [24]. Upon contacting the first Author stating interest in an interview, an information sheet and consent form was sent via email at least 48 h prior to the interview taking place, with the consent form returned via email to JR and stored separately from any data collected in order to maintain anonymity. Each interview also started with a reminder of ethical considerations, and verbal consent was gained prior to beginning the interview proper. Immediately upon study completion, a debrief form was emailed to participants. No job roles were excluded, and those interviewed included a variety of clinical and nonclinical roles such as nurses, administrators, managers, occupational therapists, and dentists. Interviews were semistructured with open-ended questions which were iterative in nature, developing through the study and expanded upon during interviews [25]. Interviews lasted on average 45 min and took place between February and May 2018. They were conducted over the telephone by JR, AM, and CB, audio-recorded, and professionally transcribed prior to analysis. The researchers were completely separate to all organisations and participants, and thus had no pre-existing relationships with any of the survey or interview respondents. Ethical approval was gained from the Bath Spa University Research Ethics Committee (JR181217).

### 2.2. Materials

#### 2.2.1. Quantitative Measures

Working conditions were measured using the 25-item version of the Management Standards Indicator Tool (MSIT) [26]. The MSIT assesses seven areas of the workplace (demands, control, managerial support, peer support, relationships, role, and change) that could lead to worsened employee and organisational outcomes if left at unacceptable levels. The 25 items are answered on a 5-point Likert scale. Higher scores equate to better working conditions. Psychological wellbeing was measured by the Warwick–Edinburgh Mental Wellbeing Scale (WEMWBS) [27]. The WEMWBS uses 14 positively phrased items to measure positive affect, psychological functioning, and interpersonal relationships (answered on a 5-point Likert scale). Higher scoring represents better mental wellbeing. Stress was measured by a 4-item version of the Perceived Stress Scale (PSS, Mind Garden, Menlo Park, CA, USA) [28]. The PSS asks how often respondents have experienced stressful situations in the past month (answered on a 5-point Likert scale), with higher scoring reflecting higher stress. Demographics (see Table 1) were also asked at the end of the survey. Participants were asked if they worked more hours than they were contracted to and to estimate how many.

Two further outcome measures (presenteeism and job satisfaction) were assessed via single-item global measures, which are argued to be as reliable as multi-item measures [29]. The job satisfaction measure asked, “taking everything into consideration, how do you feel about your job as a whole?” [30], with responses from 1 to 5 (extremely dissatisfied to extremely satisfied). Presenteeism was measured via the question, “as far as you can recall, has it happened over the previous 12 months that you have gone to work despite feeling that you should really have taken sick leave due to your state of health?” [30], and responses given on a 4-point Likert scale from ‘no, never’ to ‘yes, more than 5 times’. Lastly, one open-ended question was asked: “in one sentence, what would you do to improve working conditions in your Trust?” [16], although these responses were not analysed due to a number (approximately 34% of respondents) of nonrespondents, potentially because this question added significantly to the length of time it would take to respond to the survey. Of all of the measures used, only the PSS has previously been used in healthcare employee populations [31,32]. However, each have been used and demonstrated to be reliable with other occupational groups, such as social workers and teachers [16,33].

#### 2.2.2. Interview Materials

Thirty-three NHS employees from three Trusts in the southwest of the UK were recruited to take part in the study. The interview schedule was not piloted among healthcare professionals, but was peer-reviewed both by members of the research team and senior management in all participating Trusts. The schedule sought to investigate the stressors associated with working in the NHS, as well as approaches which can be taken in order to improve upon such stressors. Participants were therefore asked to outline any particular stressors that they faced at work: for example, “what difficulties do you experience at work that may affect your wellbeing”, as well as the resources available to buffer these stressors, such as, “how can we build upon the work that your work does well in order to support your wellbeing”. Finally, interviewees were asked for changes or interventions which could be taken in order to improve upon these stressors: for example, “what initiatives would you like to see in your job to support your wellbeing?”, while also building on the positivity associated with the resources already available at work.

### 2.3. Analytical Strategy

Quantitative data were analysed using IBM SPSS 24.0 (IBM, Armonk, NY, USA). Initially, descriptive statistics were presented for each measure (mean, standard deviation, and frequency where available), as well as comparisons to UK-wide benchmarks where possible. Subsequently, a series of regression analyses were undertaken in order to investigate the influence of working conditions on stress and psychological wellbeing.

Qualitative data was analysed by a qualitative researcher (CB) using thematic analysis following steps by Braun and Clark [25]. Following immersion into the data by repeatedly listening to the recordings, NVivo software (QSR International, Melbourne, Australia) was used to code the transcripts into themes and subthemes. Themes were created, split into subthemes, and omitted throughout analysis as they were reviewed. Half of the data was then independently coded by JR in order to reach a consensus. Verbatim quotes with labels were used to illustrate each theme.

In order to integrate the findings of the quantitative and qualitative approach into a truly mixed methods study, we utilised a triangulation protocol via the organisation-wide surveys and semistructured interviews [34]. As such, we disseminated the organisational survey to all in each of the participating organisations and analysed the results. Following the survey, we invited interested parties to take part in the semistructured interviews, while maintaining an open and exploratory interview schedule. Following analysis, the findings from each of the quantitative and qualitative elements were considered together to discover whether there was agreement or dissonance between the two sets of findings [34].

## 3. Findings

### 3.1. Survey Results

Invitation emails were sent to all employees in the three Trusts (n = 11,370), with 1644 responses gained (a response rate of 14.5%). Apart from the demographic questions, all questions were forced-response, and thus there was no missing data. The majority of participants identified working excess hours, on average ranging from 3.7 to 7.2 h excess per week. Compared to reference scores [35] of over 67,000 public and private sector employees in the UK, working conditions were relatively positive (see Table 2). These reference figures have previously been used with UK social workers [16] and teachers [33], among many other populations. However, workload demands on management, doctors, and physiotherapists and a lack of control and role understanding in physiotherapists scored in the 10th percentile, or worse than 90% of those in reference figures. Similarly, the amount of control over the way in which physiotherapists and healthcare assistants do their jobs scored in the 10th percentile, as did management and physiotherapists’ understanding of their role in the organisation. Other than this, all outcomes were in the 25th percentile or greater, with the demands faced by administrators and peer support for physiotherapists scoring in the 95th percentile.

Apart from management in this sample, scoring was above average on the PSS [36], suggesting higher stress, although still within one standard deviation. Mean scoring on the WEMWBS was 49.9 [37], higher than all employees irrespective of job role in the presented sample, indicating lower average psychological wellbeing than the UK average. Findings also demonstrate that most (59.1%) were satisfied in their jobs, whilst 42.1% had been to work at least twice in the previous 12 months despite being so ill they should have stayed off work. Interestingly, nurses and healthcare assistants were least satisfied whilst also being in the top three of presenteeism scorers.

Regression analyses (Table 3) were conducted to investigate the influence of working conditions on perceived stress and psychological wellbeing. The stress regression model demonstrated good fit, accounting for 20% of variance for all participants. Six MSIT variables significantly influenced both perceived stress (managerial support was the only one which did not; see Table 3) and psychological wellbeing (excluding demands), accounting for 27% of variance.

### 3.2. Interview Findings

Three overarching themes from the interviews were identified from the 33 semistructured interviews undertaken. These were wellbeing at work, relationships, and communication.

#### 3.2.1. Wellbeing at Work

Many contributing factors were mentioned for those experiencing work stress, including high workload and changes at work. Generally, it was felt that there was insufficient support and understanding around mental health issues, emphasizing not only the impact of poor work on individual wellbeing, but also what organizations can do to prevent employees from experiencing negative work stress and wellbeing.

Mental health at work: Participants spoke of mental health problems which resulted in being absent from work for a period, with this being attributed to work stress. Many participants described a lack of support and a lack of understanding about mental health issues from management. They proposed more mental health education for managers to increase understanding and thereby level of support, including more frequent debriefs. As an example: “If somebody has been off with stress or depression or anxiety, when they come back, the same conversations should take place…what can we do to help you get back to work and make things more comfortable for you?” (P15, nurse, female).

Workload: Workload was discussed as the predominant stressor among the majority of participants. Employees spoke of unrealistic deadlines and workloads and consecutive clinic appointments: “Sometimes you’re asked to do things at very short notice which sometimes is nigh impossible” (P16, consultant, female). This was spoken about in connection with a high level of stress: “It’s very much workload is causing the biggest stress at the moment.” (P20, male, administrator). This also led to the majority describing an inability to take any kind of a break while at work: “It seems to be a habit across my team. I see people doing it all the time. I think it’s an organisational thing. We do not take half an hour out of our day to get a breath of fresh air or anything like that” (P25, nurse, female).

Improving mental health: Some employees suggested that it would be helpful to have a room where staff could go to get away from their desk to have a break, especially during a difficult time: “I’d like the idea of a quiet room, just to sit down for five minutes. If you’ve had a difficult call, you can go in and bring yourself back in and remind yourself that you’re okay and that person’s going to be okay” (P7, administrator, female). Participants were also positive about “extra” wellbeing events which were put on by their employing organisation specifically aimed at improving wellbeing: “Get people to go into departments…and show them different ways of keeping fit or maybe dealing with stress. That sort of thing would be really good.” (P3, occupational therapist, female). However, a lack of communication meant that some respondents did not know when and where these events were happening, and many activities were hosted at organisational “hub” organisations, but not associated satellite sites such as hospitals: “There are not many days and things that are really accessible for us.” (P28, doctor, male).

#### 3.2.2. Relationships

This theme relates to relationships that employees have within work, with their line managers and peers, and the positive influence that such relationships can have on wellbeing. Peers were a source of support in this group, while there was a mix of positive and negative relationships with managers. Finally, the subtheme of supervision revealed that a number of participants felt that they would benefit from an increase in the frequency and quality of supervision from management.

Relationship with line manager: Participants had polar views when it came to their relationships with their managers. Those who participants saw in a positive light were described as being good communicators and supportive. They were understanding both in terms of managing workload and issues that may be affecting their staff personally: “Whenever I’ve needed time off personally, my manager’s been fantastic. I can’t fault her on those things, which I think staff really value, because it makes you feel like you’re a human being…you’re seen as a valuable member of staff” (P22, manager, female). Those who felt negatively towards their line manager described them as being poor communicators and having weak management skills: “They cannot manage people…they’ve had no management development” (P11, no job role recorded).

Relationship with colleagues: Employees clearly experienced a great deal of support from their team at work and were markedly positive about their relationships with their peers. They appeared to be a key source of encouragement for many interviewed. Communication was again highlighted as being important: “The communication here is amazing. It’s really as if like I’m in a big family. It’s the best place” (P24, nurse, female). Listening skills and being helpful and friendly were important: “They’re so helpful and friendly. If you’ve got an issue, they’re quite happy just to put their issues aside and listen to you and give you praise when it’s needed. They make you feel not just needed but appreciated” (P7, administrator, female). It appeared therefore that being part of a positive team environment impacted on how much they enjoyed their job.

Supervision: Many felt that both clinical and general supervision was lacking, despite being important. In some cases, supervision was missing altogether or not being carried out as frequently as it should be: “We’re supposed to do clinical supervision, and I say supposed, because it never seems to properly take off” (P32, nurse, female). Additionally, there was sometimes a reactive instead of proactive practice in providing supervision to clinically focused staff: “Clinical supervision is something that we’re supposed to do but it’s never really been ingrained in our culture…it tends to only happen when there’s been a problem” (P25, nurse, female).

#### 3.2.3. Communication

This theme focuses on both top-down and bottom-up communication. Indeed, each organisation is constantly changing due to the financial pressures currently facing the NHS [3], and the way in which these changes are communicated was described as a particular stressor.

Bottom-up communication: Of those interviewed, some felt able and motivated to put constructive suggestions for change forward to managers/senior managers; however, for many, there was negativity surrounding this issue. Some described being far too busy: “it’s all about surviving the day” (P17, doctor, male), or did not feel that their views were valued: “the impression you get is if it doesn’t come from them, it doesn’t count” (P1, nurse, female). Participants also spoke of not receiving any feedback if they did put forward ideas: “I think that when you’re constantly feeling that you’re not being listened to then that is part of the problem” (P25, nurse, female).

Top-down communication: Many spoke about poor communication from management, particularly regarding changes which were directly relevant to them, and not finding out about changes until they had actually taken place. A lack of communication and consultation about change was described as being something which affected staff wellbeing: “it’s just as if the organization at the top doesn’t actually care about how that makes people feel” (P15, nurse, female). Relatedly, staff described that information about change was not shared with them and were worried about redundancies and having to reapply for jobs. Employees wanted open and honest communication from the Trust.

## 4. Discussion

This study examined the influence of working conditions on employee wellbeing and stress in three NHS Trusts using mixed methods. Survey results suggested that working conditions performing well—better than 75% of benchmark scorers [35]—are management and peer support, and change communication within the organization. Furthermore, management respondents reported having autonomy and change communication, and the workload of those in administrative roles, at a level better than 90% of benchmark scorers. This is positive given that recent research has shown teachers [33] and social workers [16] scoring in the bottom 90th to 95th percentiles. More negatively, demands were worse than 90% of benchmark scorers in some staff groups. Similarly, over 40% were not satisfied in their job, and over 40% attended work at least twice in 12 months while ill (presenteeism). Regression outcomes demonstrate perceived stress and psychological wellbeing were influenced by a number of working conditions.

In the qualitative data, three themes emerged. The first was “wellbeing at work”, in which participants described a too-high workload, inability to take breaks during work, and changes at work contributing to a high level of stress in them and their colleagues. There was not enough support around mental health issues, but employees did welcome wellbeing events provided by the Trust and wanted easier access to these. The second theme was “relationships”, in which participants described receiving a lot of support from peers and around half also felt well-supported by managers who were emotionally supportive and easy to communicate with. It was also revealed that supervision could be improved. Finally, in “communication”, participants did not feel they had time to feed up their views to senior staff or felt their views were not valued or they would not receive feedback.

There were both agreements and dissonance between the quantitative and qualitative findings. Peer support was positive in both, potentially buffering against the negative influence of other conditions on wellbeing. Additionally, demands (which can include workload, work environment, and work patterns) was a difficulty in both the survey and interviews, with almost all describing a volume of work which was too high and a culture of consistently missing lunch breaks. However, while the survey suggested managerial support was good in those who took part, the qualitative data revealed that while half felt very supported by their manager, half did not. This finding could imply that those with problematic relationships with their manager may have been more likely to take part in the interview stage. Furthermore, while the management of and communication about change scored well in the quantitative data, communication about change appeared to be an issue which was causing a lot of stress and was described as being done poorly in the qualitative data. Finally, an understanding of one’s role in the organisation scored poorly (worse than 90% of benchmark scorers), while this did not emerge as a major issue through analysis of the qualitative data.

Together, the findings suggest that there are a number of areas which could be contributing to lower levels of mental wellbeing in NHS employees. Poor working conditions and high levels of stress at work have been demonstrated to influence organisational outcomes such as dissatisfaction at work, absenteeism, and presenteeism [16], and subsequently impact on patient care [19]. Indeed, research with domiciliary care workers [38,39] has similarly demonstrated the impact that role understanding and occupational autonomy has on individual psychological health and wellbeing. In accordance with the job demands–resources [8] model, a number of the issues discussed could be adding to the demands experienced while working in the NHS. For example, the high workload described by many, such as high caseload, high numbers of patients to be seen, or large quantity of requests for reports often in an impossible time frame, is an obvious example of this. Workload could be reduced by increasing levels of recruitment, though we recognise economic constraints are currently in place. Changes taking place which affect, or threaten to affect, employees’ work and environment directly and are a cause for concern about redundancy can also add to demands experienced, especially if the changes are not communicated well or in a timely manner. Strategies to improve communication about change are warranted. In terms of information flowing the opposite way, the data suggested that some workers felt they either did not have the time to put their ideas forward or felt their ideas were not valued. It may be useful to give members of staff more opportunities to put forward suggestions and give feedback about any changes so that staff feel their input is valued. Feeling that there is a lack of opportunity to put forward ideas and feeling undervalued as a member of staff could also act as an additional demand.

The JDR [8] model suggests that there are factors which can act as buffers to stress at work and these resources may reduce the effect of the demands placed on the individual. One such likely buffer found in both the quantitative and qualitative results was the support of colleagues. Support from peers can help by spreading workload and providing emotional support. It has been widely suggested to be a significant buffer toward the experience of stress at work [40]. On the whole, it was found to be positive in this group especially if colleagues had good communication and listening skills and were helpful and personable. In order to increase this buffering effect, it may be useful to encourage regular, formalised communication and peer support groups between team members through meetings and possibly training around good communication. Managerial support scored well in the survey, with more complex findings in the interviews. Those with a good relationship with their supervisor could benefit from this as a buffer to stress at work. Those who felt positive about this relationship emphasised supervisor who were understanding and made them feel valued. Supervisors can provide individualised support such as supervision and one-to-one meetings and can play a part in greater job role understanding (which scored poorly in the survey). In the supervision subtheme, some employees described not having enough supervision. Regular sessions for all staff could nurture relationships, promote better communication, increase feelings of being supported, and could improve wellbeing. There were other factors which could also act as buffers with some changes implemented. For example, the results suggested that there was a culture of not taking lunch breaks. A break from work, with possibly fresh air and exercise or time to talk to colleagues in a relaxed manner, could provide a buffer to daily stress. This could be encouraged by providing lunchtime walking clubs, providing suitable areas to eat away from the desk inside and out, and encouraging managers to lead by example in terms of break-taking. Staff may perform better after a break [41].

Wellbeing activities provided within the Trust could also be regarded as resources to balance out the demands employees are confronted with. The results presented here suggest that wellbeing-related activities should be made available to individuals in a variety of times and locations (in order to take into account shift patterns and the diverse geographical nature of many NHS Trusts), and possible cover for staff may be important so that they have the opportunity to attend. Also, it should be clearer what events are available to staff and ensure that this information is made available in plenty of time prior to the events. Additionally, it was suggested that a room for staff to go when they are having a difficult time for a short break would be helpful to allow individuals some space from work. Finally, more mental health awareness and promotion of counselling services available may increase uptake, which could provide a buffer to demands placed on the employees.

### 4.1. Strengths and Limitations

Exploring the issue of NHS workforce wellbeing using both quantitative and qualitative methodology is a strength of this study. This resulted in both a large data set which could be used to compare NHS employee working conditions to mean UK scores and to other public sector employees, and a richly detailed dataset in which to examine the topic of staff mental wellbeing. NHS employees from a wide range of roles from three large Trusts participated in both aspects of the research. Furthermore, the data suggested a number of practical suggestions which could be implemented with health services organisations in order to support their staff.

In terms of limitations, for the survey, there was a low and imprecise response rate. While these are often described as issues for internet-mediated studies, the approach also allows the collection and analysis of a large sample size while maintaining anonymity and confidentiality. Furthermore, the self-report and cross-sectional nature means causality cannot be ascertained. There may also be errors in self-reporting of contracted/actual hours. For the interviews, since participants were self-selecting, there may have been a degree of bias in the sample in terms of employees who were particularly stressed or who had experienced negative relationships at work. However, the sample provided an opportunity to explore potential areas for improvement.

### 4.2. Implications and Future Research

The survey results emphasised a low scoring for understanding how one’s role fits within the organization, with this contributing to both wellbeing and perceived stress scores. This deficiency could be increased through targeted information sharing and discussion during one-to-one meetings. There were some differences in roles, with management, doctors, and physiotherapists scoring particularly poorly for demands, while physiotherapists and healthcare assistants scored poorly for control. Targeted aims to reduce demands and increase the level of control in these roles may be valuable. Future research should also investigate these role differences in more depth, such as whether differing interventions would be more amenable and have greater efficacy in differing job roles.

It is clear that peer support is potentially a significant resource for employees [40]. We suggest that formal peer support groups and networks such as Schwartz Rounds be set up across NHS Trusts. Additionally, an increase in managerial support, for example by providing regular one-to-one meetings to staff, may reduce stress. Also, in the results, change communication was highlighted as lacking, and with it, an absence of consultation over change. This could be addressed by providing opportunities for employee input and regular and timely information about upcoming changes. Future research could also focus on differences in experience between managers and employees. We have demonstrated some differences between management and employees in working conditions (as per the MSIT), and thus potential differences could be investigated in more depth.

Previous researchers focusing on predominantly health behaviours have found workplace wellbeing interventions in the NHS to successfully improve physical activity and job satisfaction and lower sickness absence [42]. A focus on interventions to increase mental wellbeing in this staff group may be beneficial. An emphasis on the areas discussed, such as improving top-down (such as Chief Executive blogs and more dedicated and targeted use of newsletters), bottom-up (such as communication training and anonymous feed-up communication systems), and wellbeing-related (for example, mental health awareness training) communication; making the best use of support by scheduling regular supervision and team meetings; improving access to wellbeing activities; and promoting break-taking would be a suitable next step in research in this field. Quirk et al. [2] demonstrate some wellbeing-related approaches which can be undertaken within the NHS despite financial and workload pressures, such as having a senior management team who will support wellbeing initiatives and a supportive organisational culture, a strategic and creative approach to implementing wellbeing initiatives, and allowing key wellbeing champions within organisations to be innovative in their approach to developing and supporting wellbeing, such as working closely with external providers. A feeling of being undervalued was mentioned by a number of participants, and indeed, in an NHS staff survey, only 41% said they felt valued [43]. This may be linked with self-esteem and employee engagement [22], and therefore it may be important to focus on changing this perception. Future research should focus on longitudinal studies to determine whether findings are consistent across time, and interventional projects which seek to reduce demands should be sought.

## 5. Conclusions

To conclude, it is clear that chronic workplace stress has the potential to negatively impact both the health of employees and the wider organisation, and in the context of healthcare, also negatively impacts patient health outcomes. This study showed that while the experience of stress may be different across different roles in the healthcare sector, across the whole workforce, various working conditions negatively influenced the experience of stress. Qualitative interviews supported this, demonstrating that working relationships, communication, and perhaps most importantly, workload, were distinct influencers of stress. As such, by supporting employees and implementing interventions within the healthcare sector which seek to improve upon these working conditions, it will not only improve health outcomes for employees, but subsequently improve things for the patients that they work with.

## Figures and Tables

**Table 1:** Demographic scoring and hour disparity for all respondents, broken down by job role.

Job Role	Mean Age (SD)	Sex		Average Length in Role	Ethnicity (%)		Disability (%)		Hour Disparity *	
		Male	Female		White British	Other	Yes	No	%	Length
All Respondents (*n* = 1644 ^+^)	46.2	16.4% *n* = 263	83.6% *n* = 1364	7 Years, 9 Months	90.0%	10.0%	10.%	90.0%	56%	5.5
Management (*n* = 163)	49.3 (8.5)	31.1% *n* = 51	68.9% *n* = 110	4 Years, 10 Months	95.2%	4.8%	3.2%	96.8%	87%	7.2
Administrative (*n* = 344)	46.6 (12.1)	9.7% *n* = 31	90.3% *n* = 309	6 Years, 7 Months	94.8%	5.2%	7.9%	92.1%	34%	4.3
Doctor (*n* = 69)	46.4 (10.5)	43.1% *n* = 29	56.9% *n* = 38	11 Years, 10 Months	77.1%	22.9%	4.3%	95.7%	66%	6.2
Nurse (*n* = 364)	48.6 (9.7)	11.0% *n* = 40	89.0% *n* = 324	10 Years, 3 Months	92.7%	7.3%	3.9%	96.1%	62%	5.2
Physiotherapist (*n* = 84)	38.5 (8.9)	8.7% *n* = 7	91.3% *n* = 76	9 Years, 8 Months	85.7%	14.3%	10.6%	89.4%	60%	3.7
Healthcare Assistant (*n* = 134)	47.0 (11.2)	10.8% *n* = 13	89.2% *n* = 119	8 Years, 2 Months	87.0%	13.0%	3.8%	96.3%	53%	5.8
Other (*n* = 486)	42.75 (11.5)	15.7% *n* = 73	84.2% *n* = 408	7 Years, 3 Months	87.3%	12.7%	6.2%	93.8%	55%	2.9

* Hour disparity relates to the average number of hours contracted to work versus those actually worked. The percentage score relates to the percentage working more hours than contracted to, and the length score is the average number of hours worked in excess each week. ^+^—Total number of respondents, including the job roles named here and ‘other’ respondents. Not all respondents provided demographic information.

**Table 2:** Mean scoring on working conditions and total scoring on the Perceived Stress Scale and Warwick–Edinburgh Mental Wellbeing Scale (WEMWBS).

Job Role	Demands	Control	Managerial Support	Peer Support	Relationships	Role	Change	PSS	WEMWBS
All Respondents (Percentile)	3.40 (50th)	3.47 (50th)	3.63 (75th)	3.93 (75th)	4.35 (50th)	4.12 (10th)	3.21 (75th)	6.62	47.24
Management (Percentile)	3.14 (10th)	3.83 (90th)	3.77 (90th)	3.95 (90th)	4.39 (50th)	4.09 (10th)	3.36 (90th)	5.92	48.83
Administration (Percentile)	3.78 (95th)	3.63 (75th)	3.71 (75th)	3.96 (90th)	4.44 (50th)	4.22 (75th)	3.34 (75th)	6.55	47.10
Doctors (Percentile)	3.13 (10th)	3.46 (50th)	3.40 (25th)	3.92 (75th)	4.28 (25th)	4.05 (25th)	3.22 (75th)	6.86	48.50
Nurses (Percentile)	3.27 (25th)	3.37 (25th)	3.61 (50th)	3.94 (75th)	4.26 (25th)	4.16 (50th)	3.15 (50th)	6.41	47.95
Physiotherapists (percentile)	3.13 (10th)	3.27 (10th)	3.77 (90th)	4.04 (95th)	4.48 (75th)	3.96 (10th)	3.27 (75th)	7.07	47.22
HCAs (Percentile)	3.58 (75th)	3.16 (10th)	3.48 (50th)	3.95 (90th)	4.20 (25th)	4.18 (75th)	3.20 (75th)	6.85	46.56
Other (Percentile)	3.31 (10t)	3.41 (25th)	3.62 (50th)	3.91 (75th)	4.37 (50th)	4.02 (25th)	3.11 (50th)	6.93	46.35

HCA = Healthcare Assistant.

**Table 3:** Linear regression analyses of the impact of working conditions on the Perceived Stress Scale and Warwick–Edinburgh Mental Wellbeing Scale across all participants.

Outcome Measure	Significantly Related Factors	Coefficient Estimates	*t*	*p*	R^2^	Adjusted R^2^
Perceived Stress Scale	Demands	−0.77	−7.12	<0.001	0.20	0.20
	Control	−0.37	−3.36	<0.005		
	Peer support	−0.50	−3.17	<0.005		
	Relationships	−0.42	−3.54	<0.001		
	Role	−0.32	−2.50	<0.05		
	Change	−0.33	−2.23	<0.05		
Warwick-Edinburgh Mental Wellbeing Scale	Control	1.37	4.30	<0.001	0.26	0.26
	Managerial Support	0.85	2.26	<0.05		
	Peer Support	2.70	5.85	<0.001		
	Relationships	1.02	2.93	<0.005		
	Role	1.35	3.62	<0.001		
	Change	1.64	3.80	<0.001		

## References

[1] Lafond S., Charlesworth A., Roberts A. (2016). An analysis of NHS finances and factors associated with financial performance. A Perfect Storm: An Impossible Climate for NHS Providers’ Finances.

[2] Quirk H., Crank H., Carter A., Leahy H., Copeland R.J. (2018). Barriers and facilitators to implementing workplace health and wellbeing services in the NHS from the perspective of senior leaders and wellbeing practitioners: A qualitative study. BMC Public Health.

[3] Robertson R., Wenzel L., Thompson J., Charles A. (2017). Understanding NHS Financial Pressures: How are They Affecting Patient Care.

[4] NHS England (2016). NHS Staff Health & Wellbeing: CQUIN Supplementary Guidance.

[5] Rimmer A. (2018). Staff stress levels reflect rising pressure on NHS, says NHS leaders. BMJ.

[6] NHS Employers Stress and Its Impact on the Workplace. https://www.nhsemployers.org/retention-and-staff-experience/health-and-wellbeing/taking-a-targeted-approach/taking-a-targeted-approach/stress-and-its-impact-on-the-workplace.

[7] Health and Safety Executive Work-Related Stress. https://www.hse.gov.uk/stress/.

[8] Bakker A.B., Demerouti E., De Boer E., Schaufeli W.B. (2003). Job demands and job resources as predictors of absence duration and frequency. J. Vocat. Behav..

[9] Schaufeli W.B., Bakker A.B., Van Rhenen W. (2009). How changes in job demands and resources predict burnout, work engagement, and sickness absenteeism. J. Organ. Behav..

[10] Schaufeli W., Bakker A.B. (2004). Job demands, job resources, and their relationship with burnout and engagement: A multi-sample study. J. Organ. Behav..

[11] Annual Statistics: 2019. Work-Related Stress, Depression or Anxiety Statistics in Great Britain 2018. www.hse.gov.uk/statistics/.

[12] Chartered Institute of Personnel Development Health and Well-Being at Work. Survey Report; London, UK, 2019. https://www.cipd.co.uk/Images/health-and-well-being-at-work_tcm18-40863.pdf.

[13] Mackay C.J., Cousins R., Kelly P.J., Lee S., McCaig R.H. (2004). ‘Management Standards’ and work-related stress in the UK: policy background and science. Work Stress.

[14] Cousins R., MacKay C.J., Clarke S.D., Kelly C., Kelly P.J., McCaig R. (2004). ‘Management Standards’ and work-related stress in the UK: Practical Development. Work. Stress.

[15] Houdmont J. (2013). UK police custody officers’ psychosocial hazard exposures and burnout. POLICING.

[16] Ravalier J.M., McFadden P., Boichat C., Clabburn O., Moriarty J. (2020). Social worker well-being: A large mixed-methods study. Br. J. Soc..

[17] Rosengren A., Hawken S., Ôunpuu S., Sliwa K., Zubaid M., A Almahmeed W., Blackett K.N., Sitthi-Amorn C., Sato H., Yusuf S. (2004). Association of psychosocial risk factors with risk of acute myocardial infarction in 11,119 cases and 13,648 controls from 52 countries (the INTERHEART study): Case-control study. Lancet.

[18] Chandola T., Brunner E., Marmot M. (2006). Chronic stress at work and the metabolic syndrome: Prospective study. BMJ.

[19] Blake H., Zhou D., E Batt M. (2013). Five-year workplace wellness intervention in the NHS. Perspect. Public Health.

[20] McFadden P., Campbell A., Taylor B. (2014). Resilience and Burnout in Child Protection Social Work: Individual and Organisational Themes from a Systematic Literature Review. Br. J. Soc. Work..

[21] West M., Dawson J.F. Employee engagement and NHS performance. https://www.kingsfund.org.uk/sites/default/files/employee-engagement-nhs-performance-west-dawson-leadership-review2012-paper.pdf.

[22] Tariq S., Woodman J. (2013). Using mixed methods in health research. JRSM Short Rep..

[23] Bastian N.D., Munoz D., Ventura M., Information P.E.K.F.C. (2016). A Mixed-Methods Research Framework for Healthcare Process Improvement. J. Pediatr. Nurs..

[24] Guest G., Bunce A., Johnson L.A. (2006). How Many Interviews Are Enough?. Field Methods.

[25] Braun V., Clarke V. (2006). Using thematic analysis in psychology. Qual. Res. Psychol..

[26] Edwards J.A., Webster S., Van Laar D., Easton S. (2008). Psychometric analysis of the UK Health and Safety Executive’s Management Standards work-related stress Indicator Tool. Work Stress.

[27] Tennant R., Hiller L., Fishwick R., Platt S., Joseph S., Weich S., Parkinson J., Secker J., Stewart-Brown S. (2007). The Warwick-Edinburgh Mental Well-being Scale (WEMWBS): Development and UK validation. Health Qual. Life Outcomes.

[28] Cohen S., Kamarck T., Mermelstein R., Mermelstein T.K. (1983). A Global Measure of Perceived Stress. J. Health Soc. Behav..

[29] Dolbier C.L., Webster J.A., McCalister K.T., Mallon M.W., Steinhardt M.A. (2005). Reliability and Validity of a Single-Item Measure of Job Satisfaction. Am. J. Health Promot..

[30] Aronsson G., Gustafsson K., Dallner M. (2000). Sick but yet at work. An empirical study of sickness presenteeism. J. Epidemiol. Community Health.

[31] Shapiro S.L., Astin J.A., Bishop S.R., Cordova M. (2005). Mindfulness-based stress reduction for health care professionals: Results from a randomized trial. Int. J. Stress Manag..

[32] Birks Y., Mckendree J., Watt I. (2009). Emotional intelligence and perceived stress in healthcare students: A multi-institutional, multi-professional survey. BMC Med. Educ..

[33] Ravalier J., Walsh J. (2018). Working conditions and stress in the English education system. Occup. Med..

[34] O’Cathain A., Murphy E., Nicholl J. (2010). Three techniques for integrating data in mixed methods studies. BMJ.

[35] Edwards J.A., Webster S. (2012). Psychosocial risk assessment: Measurement invariance of the UK Health and Safety Executive’s Management Standards Indicator Tool across public and private organizations. Work. Stress.

[36] Warttig S., Forshaw M.J., South J., White A. (2013). New, normative, English-sample data for the Short Form Perceived Stress Scale (PSS-4). J. Health Psychol..

[37] Morris S., Earl C., Neave A. Health Survey for England 2016 Well-being and mental health. Health and Social Care Information Centre: Health and Social Care Information Centre, London, UK, 2018. http://healthsurvey.hscic.gov.uk/media/63763/HSE2016-Adult-wel-bei.pdf.

[38] Ravalier J., Fidalgo A.R., Morton R., Russell L. (2017). The influence of zero-hours contracts on care worker well-being. Occup. Med..

[39] Ravalier J., Morton R., Russell L., Fidalgo A.R. (2018). Zero-hour contracts and stress in UK domiciliary care workers. Health Soc. Care Community.

[40] Peterson U., Bergström G., Samuelsson M., Åsberg M. (2008). Nygren, Åke Reflecting peer-support groups in the prevention of stress and burnout: Randomized controlled trial. J. Adv. Nurs..

[41] Hunter E.M., Wu C. (2016). Give Me a Better Break: Choosing Workday Break Activities to Maximize Resource Recovery. J. Appl. Psychol..

[42] Blake H., Lee S. (2007). Health of community nurses: A case for workplace wellness schemes. Br. J. Community Nurs..

[43] Wilkinson E. (2015). UK NHS staff: Stressed, exhausted, burnt out. Lancet.

